# The Matron's Department

**Published:** 1905-11-04

**Authors:** 


### THE MATRON'S DEPARTMENT.

### XII.—THE LAUNDRY, CLEANING, ETC.

It is important to have a competent head laundrymaid to control the laundry work, and it is far better economy to pay good wages and secure a trustworthy woman than to depend on cheap labour. The financial success of the home laundry depends first on the engine-room, secondly on the organisation of the work, and thirdly on the supervision of materials. With the engine-room we are not in this place concerned, for it is not probable that it will fall to the matron's lot to superintend this department. But however competent the head of the laundry may prove, the control of the staff and of the materials used should not be allowed to pass out of the matron's hands. A very common mistake into which the institution may drift unaware is to employ too many hands in proportion to the work executed. The ordinary laundry hours are 10 hours a day and five on Saturday, and it ought to be possible to get through an average of 1,000

pieces a week for each woman employed. This supposes the use of the ordinary mechanical appliances found in modern laundries. Each woman's work should be strictly defined, and a list containing the duties to be done every day in the week should be compiled by the aid of the head laundry-maid, and after due trial should be regularly adhered to, each woman receiving a copy of her own orders. We quote a specimen day in a hospital laundry where two resident laundry-maids and four women by the day are employed.

*Monday.*

*No.* 1 *Laundress.*—Books; sort doctors' washing; help sort nurses' washing; afternoon, drying table-linen and aprons.

*No.* 2 *Laundress.*—Sweep and dust laundry every morning; make beds and tidy laundresses' room; help sort nurses' washing; afternoon, drying with laundress No. 1.

*Women.*—No. 1.—Sort house and nurses' linen; mangling.
No. 2.—Fold ward work; drying ward work till noon; fold ward work and nurses' aprons; take books to wards every night 6.45.
No. 3.—Sort ward work every morning; wash flannels, etc.; starch table linen.
No. 4.—Drying morning; folding ward work for the rest of the day.

The quantity of soap, soda, etc., for the laundry should be issued once a week to the head laundry-maid, and what is not in immediate use she should keep locked up. The amount required will vary slightly from week to week, and much will depend on the quality of the soap in use. An inferior quality contains an undue proportion of water, and is hence very wasteful. There can be no doubt that the standard of work done in the laundry will correspond to the interest taken in it by the matron, and her own knowledge of what can and ought to be achieved. Her inspection will be valuable in proportion to her appreciation of the difficulties to be overcome, and her criticism will be more sensibly felt if her approbation of good work is sometimes cordially expressed.

The items of cleaning and chandlery, and of hardware, brushes, crockery, etc., are each responsible under their respective headings in the Uniform System of Accounts for expenditure, varying between 15s. and 25s. per available bed. These articles, cheap, in common use, under the control of subordinate workers, are peculiarly exposed to the danger of waste. Many housekeepers hardly attempt any check in this direction. Their maxim is that "servants must have what they want for cleaning purposes," and they are thus left with only the empty resource of grumbling when waste occurs. It is certainly very bad policy to be suspicious and grudging in the issue of necessary stores, but it is quite possible in a household where week by week the same amount of cleaning has to be gone through, to arrive at a reasonable average of requisites for the purpose, and until this average has been ascertained, no real control can be exercised. The housekeeper must find out for herself by consultation with trustworthy subordinates what is a fair allowance, and must issue to each person individually her own share. The mere fact of receiving from the hands of the housekeeper once a week a given weight of soda or soap for her own use impresses the worker with the value of the article and gives a sense of being trusted, altogether thrown away when all use from a common stock. A girl grows to understand under this system that she will get credit for care and will soon develop the sense of responsibility which distinguishes the trained from the untrained servant. The housekeeper must see that each one has a shelf allotted to her for her own materials and proper receptacles to hold them, and will be well rewarded for attention to these little details by the saving effected. She should point out the importance of saving in little things when occasion serves, and the fact that waste in a hospital is waste of money given for the poor.

We quote a list of such stores "to be given out by the housekeeper every Friday" as it has come under our hands by the courtesy of the matron who compiled it.

| — | Kitchen. | Housemaid. | Doctor's-maid. | Kitchen-maid. | Four Yard Porters (each). | Engineer. | Museum Boy. | Four Ward Scrubbers by Forewoman. | Total. |
|---|---|---|---|---|---|---|---|---|---|
| Soda (lb.) .. .. .. | 20 | 1 | 1 | — | — | — | 2 | 25 | 49 lb. |
| Soap, yellow (3-lb. bars) .. | 2½ | ⅓ | ⅓ | — | — | 2½ | ⅓ | 3 | 9 bars |
| Sanitas (1-lb. bars) .. .. | — | ½ | 1½ | ½ | ½ | 1 | ¾ | — | 4¾ |
| Hudson's (1-lb. pkt.) .. .. | 2 | * | 1 | 1 | — | — | 1 | — | 5 |
| Monkey soap (cake) .. .. | 1 | ½ | ½ | ½ | — | — | ½ | — | 3 |
| Tapers .. .. .. .. | 3 | — | 1 | — | 1 | 2 | 1 | — | 8 |
| Matches (small boxes) .. | 4 | 3 | 12 | 2 | 2 | 6 | 3 | — | 32 |
| House flannels (7 doz.) .. | 1½ | † | † | — | — | — | ‡ | 4 | 5½ |
| Emery cloth (sheets) .. .. | 1 | 1 | 1 | 2 | — | † | 1 | — | 6 |
| Blacklead (pkts.) .. .. | 2 | 2 | 2 | — | — | — | — | — | 6 |
| Etc., etc... .. .. .. | — | — | — | — | — | — | — | — | — |

* Occasionally. † As required. ‡ If required.

This list does not of course exhaust either the list of materials or of those to whom they are distributed, but is inserted as a sample of the careful estimates which tend to economy. A similar list of requirements is made out for all the wards according to size, and the matron is by this means enabled to tell at a glance exactly what has been used, by whom and for what purpose out of even such elusive articles as matches and tapers.

It is necessary to keep the stock of brushes and brooms under constant inspection to see that they are in proper condition and renewed when they are worn out, and the housekeeper should not omit to check them over at least once a month in each ward and in the kitchen premises. The worn-out article should in every case be produced before a new one is issued. The following should be the ward stock of brooms and brushes:—Two long-handled brooms, feather broom, soft-haired broom, set of stove brushes, set of shoe brushes, pail brush, sweep brush, two small dusting brooms.

An important feature in the smooth working of an institution is the store-room, in which a stock of all articles commonly used is kept in reserve. It is always better in a regular household to limit the number of breakable articles in use to the number actually required, with one or two over, but it is very undesirable to be obliged to send out to buy whenever a tumbler or cup is broken, and even in the smallest household the housekeeper must have reserves. The stock-room should be used for no other purpose, and the key should be retained by the matron or her delegate, who should supply goods only on orders signed by the Sister of the ward or other authority, and initialled by the matron. A stock-book, containing an inventory of all the articles should be kept and nothing should be allowed to run quite out of stock. Once a quarter or twice a year, as may be found convenient, the stock should be replenished.

There is a great difference in the amount of things worn out and broken as between one ward and another, and nothing quickens the sense of responsibility better than to bring this under notice by a regular system of reports. The report which should be circulated at regular intervals is compiled from the signed requisitions for household articles described above. The report would be somewhat as follows, specifying merely the articles issued during the previous quarter and by whom requisitioned:—

*Quarter ending*............................

| — | Sister. | Sister. | Sister. | Sister. | Total. |
|---|---|---|---|---|---|
| Tumblers ... ... | | | | | |
| Feeding-cups ... ... | | | | | |
| Basins ... ... ... | | | | | |
| Teapots ... ... | | | | | |
| Plates ... ... ... | | | | | |
| Brooms... ... ... | | | | | |
| Gas globes ... ... | | | | | |
| Etc., etc. ... ... | | | | | |

Thus, without any complaint or unpleasantness, merely in the ordinary course of business, the sins of the lax sister are made public, and a spirit of carefulness is infused into each ward.

